# Letter to the Editor on “A Randomized, Controlled Trial of Exercise for Parkinsonian Individuals With Freezing of Gait”

**DOI:** 10.1002/mds.28294

**Published:** 2020-11-20

**Authors:** Jorik Nonnekes, Moran Gilat, Nicholas DʼCruz, Bauke W. Dijkstra, Bastiaan R. Bloem, Alice Nieuwboer

**Affiliations:** ^1^ Radboud University Medical Centre; Donders Institute for Brain, Cognition and Behaviour Department of Rehabilitation Nijmegen the Netherlands; ^2^ Department of Rehabilitation Sciences Neurorehabilitation Research Group (eNRGy), KU Leuven Leuven Belgium; ^3^ Department of Neurology Radboud University Medical Centre, Donders Institute for Brain, Cognition and Behaviour Nijmegen the Netherlands

**Keywords:** freezing of gait, Parkinson's disease, rehabilitation, excercise

In a recent issue of *Movement Disorders*, Silva‐Batista and colleagues reported the results of a randomized, controlled trial on adapted resistance training with instability (ARTI) compared with traditional motor rehabilitation (TMR) in people with Parkinsonʼs disease (PD) and freezing of gait (FOG).^1^ The authors found that ARTI significantly improved several outcomes of symptom severity, including FOG, compared with TMR. Nonpharmacological interventions are essential in managing FOG, and we commend the authors for aiming to increase the scientific evidence in this field. We further realize such intervention studies take tremendous amounts of effort to conduct. However, we are concerned about the safety of the reported training and about several methodological issues, which may cloud accurate data interpretation.

Both groups contained a select group of unusually severe freezers based on NFOG‐Q scores at baseline (TMR, 22.3 ± 6.0; ARTI, 21.6 ± 5.7), some of whom must have had FOG episodes lasting >30 seconds.^2^ Moreover, the Hoehn and Yahr stages in both groups ranged from 3 to 4, indicating a moderate to severe degree of postural instability. Considering this severe level of disability, our concern relates to the requirements and feasibility of the ARTI intervention in such an extremely fall‐prone cohort. Patients were asked to perform high‐level balance exercises, that is, weighted lunges and squats on unstable surfaces, which could lead to falls and should therefore not translate to self‐administration without expert supervision. Notably, adherence rates were exceptionally high (100% for 612 ARTI sessions) and adverse events scarce for such high‐risk activities, although it remains unclear how these were monitored.

The primary outcome was the New Freezing of Gait Questionnaire (NFOG‐Q). Recent work comparing consecutive measurements in 2 independent PD populations without intervention showed that the NFOG‐Q has a minimal detectable change (MDC) of 9.95 points.^3^ This is larger than the reported effect of ARTI (4.7 points). The authors did not refer to this work, but calculated the MDC using pre‐ and postassessments of their own active control intervention (2.7 points). Thus, although the ARTI intervention seemed to significantly improve several outcomes, the conclusion that the intervention effectively reduced FOG is likely confounded by test‐retest error.

Furthermore, the FOG ratio, an objective measure of FOG severity, strongly deteriorated in controls (12.8 preintervention vs 20.9 postintervention), which is unexpected during this brief trial duration (3 months), particularly because controls received an active intervention. This unexplained worsening in controls may have inflated the effect of the ARTI intervention on this outcome. Also, participants had abnormal FOG‐ratio values (average of 20.9), whereas they were ON medication, which is much higher than those reported in patients OFF medication in the initial FOG‐ratio validation article (0‐13).^4^

Inconsistencies also exist between the trial registration and the reported outcome measures, including those for gait, balance, and fMRI analyses, hinting toward selective reporting. Further, the statistical analyses did not account for baseline performance, although the TMR group seemed to have scored worse on multiple metrics. Last, the original trial registration targeted a sample of n = 32 (albeit without power calculation), but it is unclear why the investigators proceeded to include n = 40.

Given these issues, we caution the clinical interpretation of this article. PD patients with FOG typically have severe balance impairment, which greatly amplifies their fall risk.^5^ The described intervention seems hazardous, based on images provided in the supplementary material, whereas the efficacy for reducing FOG appears uncertain. Therefore, we believe that caution and further studies are warranted before advocating the ARTI intervention for people with FOG.
